# Evaluating the Time Interval Between Symptoms Onset, Diagnosis, and Therapeutic Intervention in Lung Cancer: A Cross‐Sectional Study in Southern Iran

**DOI:** 10.1002/cnr2.70026

**Published:** 2024-10-18

**Authors:** Alireza Salehi, Alireza Rezvani, Mohammad Javad Fallahi, Ghazal Gholamabbas, Maryam Moayedfar

**Affiliations:** ^1^ Department of MPH, School of Medicine Shiraz University of Medical Sciences Shiraz Iran; ^2^ Thoracic and Vascular Surgery Research Center Shiraz University of Medical Sciences Shiraz Iran; ^3^ Shiraz Nephro‐Urology Research Center Shiraz University of Medical Sciences Shiraz Iran; ^4^ Internal Medicine Department Shiraz University of Medical Sciences Shiraz Iran

**Keywords:** diagnostic delay, Iran, lung neoplasms

## Abstract

**Background and Aim:**

Delay in diagnosis and treatment of lung cancer is thought to be a major cause of its poor outcomes. We evaluated the delays within the presentation to the initiation of diagnostic and therapeutic interventions amongst lung cancer patients in Southern Iran.

**Methods:**

This cross‐sectional study was conducted from March 2019 to March 2021. The data collected through interview included socio‐demographic, medical and clinical findings, and the time intervals needed to visit physician, refer to specialist, request diagnostic procedures, reach diagnosis of lung cancer, and hospitalization.

**Results:**

Eighty‐nine patients (58 males and 31 females) with a mean age of 61.01 ± 12.25 years were included. The median time of symptom presentation and first physician visit interval was 25 days. Sixty‐five days were spent for requesting, performing, and evaluating the diagnostic procedures. The median interval between diagnosis and initiation of treatment was 16 days. Totally, it took an average of 122 days from the presentation to the definite diagnosis of lung cancer. Patient‐, diagnosis‐, and treatment‐related delays were not significantly correlated with any of the demographic, socioeconomic, and clinical (disease stage, symptom) variables, as well as the diagnosis tool and the first physician who visited the patient (*p* > 0.05).

**Conclusions:**

There was a significant delay but relatively similar to other countries in the diagnosis and treatment of lung cancer patients in Southern Iran. The largest portion of delay could be attributed to the raising clinical suspicion in the physicians, referral for diagnostic assessments, and the diagnosis process.

## 1 Background

Lung cancer is the leading cause of cancer‐related mortality in men and second in women worldwide [1]. As a core concept in all cancers, prevention is the primary intervention against lung cancer. Currently, in addition to smokers, lung cancer is observed in non‐smokers and ex‐smokers, and our knowledge and ability are still insufficient to effectively prevent lung cancer [2, 3]. Due to the lack of public health initiatives for smoking cessation and lower access to health facilities, the incidence of lung cancer is increasing in our country as other low‐ to middle‐income countries [4, 5].

Despite the use of various strategies and programs such as awareness programs, low‐dose CT screening, targeting high‐risk groups, the prognosis of lung cancer is still poor. For example, only 17.7% of women and 12.9% of men in the UK survive for 5 years or more after the diagnosis of lung cancer [6, 7, 8, 9, 10, 11, 12, 13, 14]. Hanna et al. [15] reported that even a 4‐week delay in cancer treatment was associated with higher mortality rate during surgical, systemic, and radiotherapy treatment for seven different types of cancer, including lung cancer. Moreover, a study on the effects of time from diagnosis to treatment (TTI) on survival in patients with non‐metastatic non‐small‐cell lung cancer demonstrated that increased TTI was independently associated with poorer survival [16]. Thus; it shows the importance of reducing the time interval between the onset of symptoms to therapeutic intervention.

The path from lung cancer diagnosis to initiating treatment can be a complex, delayed, and time‐consuming process (determination of operability vs. resectability, need for expensive PET imaging, invasive mediastinal staging), which is presumed as one of the main factors related to the high frequency of advanced disease at diagnosis [17]. Another approach is to focus on reducing the avoidable delay within the presentation‐to‐treatment interval. In this respect, several guidelines have been implemented in some countries. The “Two‐Week Wait appointment system” of the British National Health Service (NHS) recommends that referral and clinical decision‐making by the general practitioner (GP) should lead to starting treatment within 31 and 62 days, respectively [17]. Moreover, in Australian optimal care pathway for people with lung cancer, recent guidelines recommend 14‐day intervals from the initial GP referral to specialist visit and from diagnosis to the first specific anti‐cancer treatment [18, 19, 20]. However, many studies have shown that this clinical pathway is often delayed, taking up to 6 months [21].

Time delays for the diagnosis and treatment of lung cancer include delays related to the patient (late presentation by symptomatic patients), the health professionals, and the health system [22, 23, 24]. In this study, we sought to investigate various time intervals from the onset of symptoms to the initiation of treatments amongst lung cancer patients referred to the lung cancer clinics of Shiraz, Southern Iran.

## 2 Materials and Methods

This cross‐sectional study was conducted on the patients with lung cancer who were referred to lung cancer clinics of Faghihi Hospital, Amir Oncology Hospital and Motahari Clinics, as well as the hospitalized lung cancer patients affiliated to the Shiraz University of Medical Sciences, South of Iran, from March 2019 to March 2021. Patients who were over 18 years of age were diagnosed with primary lung cancer using pathological assessments, were diagnosed within the last 3 months, and had no prior cancer history were enrolled in the study using convenient sampling. The patient was excluded if his outcome measures could not be retrieved. The protocol of the study was approved by the Ethics Committee of Shiraz University of Medical Sciences (code: IR.SUMS.REC.1398.208).

A data collection form was developed by the research team, which included demographic data, medical and clinical findings, and information about the days and time intervals needed by lung cancer patients to visit a physician, refer to a specialist, request diagnostic procedures, reach diagnosis of lung cancer, and hospitalize. The patients' education level was categorized into illiterate, diploma or lower (12 or less years of schooling), and higher than diploma (associate degree and higher). Patient's income was simply categorized into low, medium, and high based on the patient's subjective viewpoints. Informed consent was obtained from the patient or their spouse or offspring. The data collection form was filled out for each eligible patient by researcher‐administered questionnaires. Statistical analysis was carried out using SPSS (SPSS Statistics Inc., Chicago, US) version 26.0. Qualitative and quantitative variables were reported using frequency (percent) and mean ± standard deviation (SD) or median (interquartile range [IQR]), respectively. Because our data were not normally distributed, the Kolmogorov–Smirnov and Shapiro–Wilk tests were used to assess the variables' distribution; then, non‐parametric tests including Kruskal–Wallis and Mann–Whitney *U* tests were used to compare the time intervals according to the demographic and medical and clinical characteristics of lung cancer patients. A *p* value < 0.05 was considered significantly different.

## 3 Results

Eighty‐nine patients were included in the study (58 males and 31 females, mean age of 61.01 ± 12.25 years). Due to COVID pandemic, referral to lung cancer clinic was reduced and recruitment was decreased, so we could not reach the predefined sample size. Most of the patients had high school diploma or lower education (> 90%), were married (> 84%) and had low income, according to their viewpoint (> 60%). Patient' demographic and clinical characteristics are summarized in Table 1 and Table S1.

**TABLE 1:** Demographic characteristics.

Variable	Frequency (%)
Age	
Mean ± SD	61.01 ± 12.28
< 40 years	4 (4.5)
40–69 years	64 (71.9)
> 69 years	21 (23.6)
Sex	
Male	58 (65.2)
Female	31 (34.8)
Marital state	
Married	75 (84.3)
Single + widow	14 (15.7)
Educational level	
Illiterate	36 (40.4)
Until diploma	34 (38.2)
Diploma and higher	19 (21.3)
Income	
Low	60 (67.4)
Medium	25 (28.1)
High	4 (4.5)

Abbreviation: SD, standard deviation.

The most common types of lung cancers were adenocarcinoma (40.4%), squamous cell lung cancer (24.7%), and small cell lung cancer (19.1%). Most of the patients were diagnosed at advanced stages (27.0% IVa and 53.9% IVb). Details of lung cancer diagnosis and staging are shown in Table S2.

More than half (58.4%) of the patients visited a general practitioner as their first physician because of a chief complaint, with a median symptom presentation—visit interval of 25 days (IQR: 7–60). In addition, 63 (IQR: 31–124) and 113 (IQR: 55–161) days were required to perform the first chest CT and tumor biopsy since the symptom presentation, respectively. Moreover, 122 (IQR: 62–169) days were required for the diagnosis of lung cancer, comprising 65 (IQR: 32–188) days for requesting, performing, and evaluating the diagnostic procedures. Furthermore, a median of 16 (IQR: 9–27) days was the waiting interval to admit in a specialized hospital ward (Table 2).

**TABLE 2:** Days and time intervals needed to visit a physician, request diagnostic procedures, diagnose lung cancer, and hospitalize amongst lung cancer patients.

Time interval (patients number)	Median [IQR]^a^
Presentation—Visiting the first physician (89)	25 [7–60]
Refer to an internist (45)	10 [4–50]
Internist to a pulmonologist (15)	10 [4–30]
Visiting a general practitioner—Visiting a pulmonologist (64)	30 [8–76]
Refer to a pulmonologist by a GP (15)	20 [3–60]
Presentation—Performing chest roentgenography (68)	60 [20–110]
Presentation—Performing chest CT (89)	63 [31–124]
Performing chest CT—Performing tumor biopsy (89)	15 [7–54]
Presentation—Performing tumor biopsy (89)	113 [55–161]
Time needed to report initial pathology reports (89)	3 [2–8]
Requesting, performing and evaluating diagnostic procedures (89)	65 [32–188]
Waiting interval to admit in a specialized hospital ward (89)	16 [9–27]
Presentation—Diagnosis of lung cancer (89)	122 [62–169]
Presentation—Start of treatment (89)	138 [86–201]

Abbreviation: IQR, interquartile range.

^a^ No normal distribution for all of the parameters (*p* Kolmogorov–Smirnov < 0.0001).

No statistically significant correlation was found between the “presentation—visiting the first physician” interval, “presentation—diagnosis of lung cancer” interval or “waiting interval to admit in a specialized hospital ward,” and demographic, medical or clinical variables, except a significantly lower time needed to diagnose the lung cancer since the presentation amongst married and single, or widowed patients (129 [IQR: 81–183] and 192 [IQR: 97–316.5] *p* = 0.041) (Table 3).

**TABLE 3:** Correlation of demographic, medical, and clinical variables with the time intervals from presentation to the treatment of lung cancer.

Variable	Presentation—Visiting the first physician		Presentation—Diagnosis of lung cancer		Waiting interval to admit in a specialized hospital ward	
	Median [IQR]	*p* ^a^	Median [IQR]	*p* ^a^	Median [IQR]	*p* ^a^
Sex						
Male	30 [7–63.75]	0.304^b^	124.5 [86–199.5]	0.914^b^	12.5 [12–27.25]	0.210^b^
Female	18 [7–60]		111 [77–204]		16.5 [8–28.5]	
Age						
< 40 years	6 [3.25–114.5]	0.268	125.5 [98.75–296]	0.875	24 [3.75–42.75]	0.793
40–69	30 [10–60]		122 [86–196]		16 [9–25]	
> 69	7 [3–90]		110 [81–214]		18 [12–30]	
Marital state						
Married	30 [7–52]	0.843^b^	129 [81–183]	0.041^b^	16 [10–27]	0.166^b^
Single + widowers	18 [7–60]		214 [97–316.5]		11.5 [7–27]	
Educational level						
Illiterate	20 [3.25–30]	0.071	98 [65.250–230]	0.315	13 [10–25]	0.789
Until diploma	30 [7.63–75]		107 [80.75–172.5]		16 [12–24.5]	
Associate degree and higher	60 [10–120]		130.5 [113–225]		18.5 [7–38]	
Income						
Low	20 [7–60]	0.245	109 [82.25–196]	0.864	14 [9–23]	0.212
Medium	60 [9–82.5]		135 [110–220]		21 [9–49]	
High	17 [3–30]		101.5 [59–143]		20 [13–24]	
Stage						
< IIIb	58.5 [7.5–60]	0.871	107 [103.5–243]	0.894	15 [10.5–33.5]	0.920
IVa	25 [7–30]		117.5 [82.25–218.25]		17 [12–24.25]	
IVb	20 [7–71.25]		124 [81.25–191.25]		13.5 [8.25–28.75]	
Presenting symptom						
Coughing	30 [10–60]	0.281	117 [80.25–248.5]	0.956	—	—
Dyspnea	27.5 [6.26–60]		116.5 [91.5–181.5]			
Hemoptysis	12 [5.75–157.5]		146 [93.5–335.75]			
Chest pain	7 [2–30]		115 [90.75–264]			
Anorexia	60 [10–60]		108 [81–194]			
Other	30 [3–90]		126 [89–178]			
Diagnosis tool						
Bronchoscopy	—	—	113.5 [85.5–192]	0.381	—	—
CT‐guided biopsy			131 [86–204]			
Surgery			110 [70.25–295.5]			
Lymph node biopsy			84 [78–162.25]			
First physician visited						
General practitioner	—	—	—	—	16 [10–25.5]	0.920
Internist					14 [8–43.75]	
Pulmonologist					20 [14–22]	
Other physicians					14.5 [4–34.5]	

^a^ Kruskal–Wallis test.

^b^ Mann–Whitney *U* test.

Abbreviation: IQR, interquartile range.

Thirty‐nine and fifty patients were presented before and after COVID‐19 pandemic, respectively. There was no significant difference in several comparisons of time intervals from presentation to the treatment of lung cancer before and after the COVID‐19 pandemic (Table 4).

**TABLE 4:** Comparison of the time intervals from presentation to the treatment of lung cancer before and after the COVID‐19 pandemic.

Interval, median [IQR]	Before	During	*p* ^a^
Presentation—Visiting the first physician	30 [83]	27.5 [53]	0.768
Visiting the first physician—Diagnosis	13 [18]	16 [17.75]	0.859
Presentation—Diagnosis	126 [94]	106.5 [98.5]	0.261
Time needed for diagnostic procedures	71 [67]	44 [58.75]	0.059

^a^ Mann–Whitney test.

Abbreviation: IQR, interquartile range.

## 4 Discussion

The main findings of the present study can be summarized as follows: a median delay of 25 days between the onset of symptom and initial consultation with the first physician, usually GPs, and an average of 65 days dedicated to diagnostic assessment were observed. It took an average of 122 days from the appearance of symptoms to confirm lung cancer diagnosis, with a 16‐day delay in treatment initiation. Consequently, the median time from presentation to initiation of treatment was 138 days. Notably, no significant association was found between these time intervals and demographic, medical or clinical characteristics or the COVID‐19 pandemic.

**FIGURE 1:**
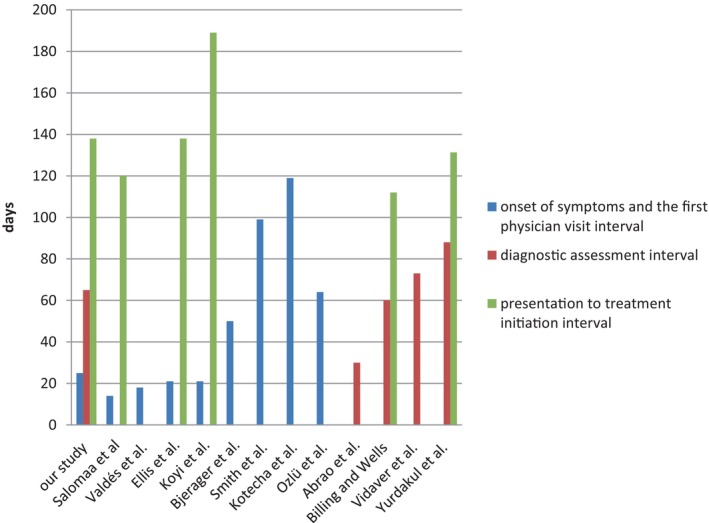
Comparison of time intervals of presentation to first physician visit, diagnostic assessment and presentation to treatment in our study and other studies.

In our study, the median time between the onset of symptoms and the first physician visit was 25 days, which was higher than reports by Salomaa et al. [24] and Valdés et al. [25] which were 14 and 18 days, respectively. However, it was close to Ellis and Vandermeer [21] and Koyi, Hillerdal, and Brandén's [26] studies which were 21 days. Our median time was less than those reported in the studies by Bjerager et al. [27] (32.5 days), Yurdakul et al. [28] (50 days), Smith et al. [29] (99 days), Kotecha et al. [30] (119 days), and Ozlü, Öztuna, and Gamze [31] (64 days) (Figure 1).

The median intervals from the GP's referral to an internist and a pulmonologist were 10 and 20 days in this study, respectively. The Australian guideline recommends a maximum of 14 days from the initial GP referral to specialist visit [20]. In addition, according to the British Thoracic Society (BTS) Standards of Care Committee, GPs should immediately refer the patients who are highly suspicious of lung cancer to a specialist, and patients should be visited by a specialist within 1 week [32]. Overall, our finding was more consistent with the UK and Australian NHS guidelines compared to that of BTS. Furthermore, in a systematic review by Olsson, Schultz, and Gould [33], on 10 studies published since 1995, it was shown that the time spent from the GP's referral to a specialist varied from 1 to 12 days.

In addition, the median time from the patient's first medical visit to performing the specialized examinations was 30 days. Sulu et al. [34], Lyratzopoulos et al. [35], and Baughan, O'Neill, and Fletcher [36] reported 40.3, 14, and 12 days for the physicians' referral delays, respectively. A critical aspect of physician‐related delay involves their referral process [18]. GPs often adopt a “watchful waiting” approach in a routine practice, anticipating that patient will return to the clinic if their symptoms persist or worsen. However, patients with cancer, for example head and neck cancer, usually do not have a second visit or are not followed up after the first visit at the primary care level. Therefore, these patients are at increased risk for delay [37]. On the other hand, excess GP visits and referrals prior to specialist's visit have been listed as another reason for such delay amongst lung cancer patients [38]. Furthermore, in a survey of 2795 GPs, Nicholson et al. [39], showed a substantial international dispersion in adherence to lung cancer guidelines. This variation in adherence to guidelines could lead to fewer requests for definitive diagnostic and therapeutic procedures, thus contributing to a delay in the diagnosis and treatment of lung cancer. In this regard, failure to request and perform chest radiography, especially in cases of chronic lung complaints, is a common malpractice [40]. Also, physicians may focus on other differential diagnoses and may not even consider the diagnosis of lung cancer, followed by the unnecessary or incorrect diagnostic procedures [41]. In general, it appears that one of the possible approaches to reducing the delay in diagnosis of lung cancer would be educational interventions in physicians and health workers at the primary care level. One potential solution to mitigate diagnostic delay and improve lung cancer management is the implementation of a fast track (e.g., patient‐centered) model [41, 42]. The aim of this approach was to simplify the patient's access to pulmonologist or oncologists through family physicians' help. It is worth mentioning that we have recently established a lung cancer registry based on our regional lung cancer collaborative clinic to further optimize care and ensure that patients receive timely and appropriate treatment [43].

An important influencing factor in delaying the initiation of lung cancer treatment is the time taken to complete diagnostic investigations. Patients usually consult several specialists and perform numerous assessments as ordered by the physicians, which leads to further delay [21]. Another finding of our study was the median time of 65 days for requesting, performing, and evaluating diagnostic procedures until the diagnosis of lung cancer, which was higher than that reported in Abrao et al.'s study with 30 days, close to Billing and Wells' study by 60 days and less than Vidaver et al. and Yurdakul et al.'s reports with 73 and 88 days, respectively [25, 28, 44, 45]. Also, our finding was close to the 62‐day interval suggested by the “Two‐Week Wait appointment system” of the UK NHS [17] (Figure 1).

The waiting interval to admit in a specialized hospital ward was 16 days, which was compatible with the intervals—from diagnosis to initiation of the first cancer‐specific treatment—recommended by the NHS England “two‐week waiting” system and Australian standard guidelines [17, 18, 19]. In addition, the cumulative time was 138 days, which compared to the studies by Salomaa et al. [24] with 120 days and Billing and Wells [45] with 112 days was higher. Our result was in the same line with Yurdakul et al. [28] and Ellis and Vandermeer's [21] findings with 131.3 and 138 days, respectively. However, the cumulative time in this study was less compared to Koyi, Hillerdal, and Brandén's [26] study with 189 days (Figure 1).

Factors such as age, gender, socio‐economic status (i.e., occupation and living place), education level, and early‐stage lung tumors can be correlated to the delay time intervals [28, 46]. However, in our study similar to a systematic review by Forrest et al. [47], none of the delay intervals was associated with demographic, medical, or clinical characteristics of lung cancer patients except marital status. Although the influence of marital status on early cancer detection was shown in previous studies, we acknowledge that our study was too limited to detect such association; it is suggested that the marital status correlation need to be validated in large studies.

It is essential to acknowledge the noticeable variations that exist in health care systems across countries, particularly between high‐income and middle‐low income countries. These variations can contribute to remarkable differences in referral and diagnosis time intervals and consequently affect patient outcomes [48].

Last but not the least, a breakout, epidemic, or pandemic (i.e., COVID‐19) can massively reframe a health care system, including redistribution of health service infrastructures (health workers, equipment, and facilities), financial barriers, lockdowns, fear of being infected in medical centers, and so forth. These changes might be led to a reduction in health service coverage, especially in low‐to‐middle‐income countries [49]. However, in this regard, in our study there was no significant difference in patient‐related delay, diagnosis, and initiation of lung cancer treatment before and after the COVID‐19 pandemic.

The present study had at least three major limitations. First, the data were retrospectively collected, raising the possibility of recall bias. Second, the patients' survival was not assessed; hence, we could not investigate the correlation between the delay intervals and patients' survival. And third, because of the small sample size, we could not detect the patient's demographic/clinical variables responsible for various delays in lung cancer diagnosis. Also, due to the significant heterogeneity in local, national, and international health systems, our results are not generalizable.

Overall, there is a significant delay in the diagnosis and treatment of lung cancer patients in Southern Iran, like some other countries. Despite the limited population size, our findings might allow an initial assessment of the extent and causes and serve as a guide for establishment of educational interventions for the public, physicians, and health workers. It appeared that the largest proportion of delay in lung cancer diagnosis and treatment was related to raising the clinical suspicion in the physicians, referral for diagnostic assessments, and the diagnosis process. On the other hand, since interventions to reduce the patient's delay may be complex, the research on primary health care components can be another solution.

## Author Contributions

**Alireza Salehi:** conceptualization, methodology, formal analysis. **Alireza Rezvani:** conceptualization, methodology. **Mohammad Javad Fallahi:** conceptualization, methodology, supervision, formal analysis. **Ghazal Gholamabbas:** writing – review and editing, writing – original draft, formal analysis. **Maryam Moayedfar:** writing – original draft, investigation.

## Ethics Statement

The protocol of this study was approved by the Ethics Committee of Shiraz University of Medical Sciences (code: IR.SUMS.REC.1398.208), and informed consent was obtained from all participants or their legal guardian(s).

## Conflicts of Interest

The authors declare no conflicts of interest.

## Supporting information


**Table S1.** Clinical and medical characteristics.
**Table S2.** Diagnosis and treatment characteristics.

## Data Availability

The data that support the findings of this study are available on request from the corresponding author. The data are not publicly available due to privacy or ethical restrictions.
