# Supplementary material for: Evaluating the Time Interval Between Symptoms Onset, Diagnosis, and Therapeutic Intervention in Lung Cancer: A Cross‐Sectional Study in Southern Iran
Source: Cancer Rep (Hoboken). 2024 Oct 18;7(10):e70026. doi: 10.1002/cnr2.70026 (PMC11488750; doi:10.1002/cnr2.70026)
Supplement: Supplementary file 1 — Table S1. Clinical and medical characteristics. Table S2. Diagnosis and treatment characteristics. [file CNR2-7-e70026-s001.docx]

**Supplementary files**

| **Supplementary table 1.** Clinical and medical characteristics | |
| --- | --- |
| Variable | Frequency (%) |
| Comorbidity  Chronic obstructive pulmonary disease  Hypertension  Diabetes, type II  Hyperlipidemia  Ischemic heart disease  Other malignancies  Others ^1^ | 48(53%)  17 (19.1)  9 (10.1)  8 (9.0)  8 (9.0)  3 (3.4)  9 (10.1) |
| Smoking  Cigarettes  Water-pipe  Both  Ex-smoker  Non-smoker | 41 (46.1)  15 (16.9)  8 (9.0)  2 (2.2)  23 (25.8) |
| Family history of lung cancers  First degree relative  Second degree relative | 6 (6.7)  3 (3.4) |
| Presenting symptoms at first medical visit  Cough  Anorexia  Dyspnea  Chest pain  Hemoptysis  Weight loss  Others ^2^ | 44 (49.4)  11 (12.4)  10 (11.2)  10 (11.2)  6 (6.7)  5 (5.6)  3 (3.4) |
| Presenting symptoms at hospitalization  Cough  Dyspnea  Weight loss  Fatigue  Anorexia  Chest pain  Hemoptysis  Hoarseness | 75 (84.3)  61 (68.5)  59 (66.3)  59 (66.3)  51 (57.3)  50 (56.2)  38 (42.7)  15 (16.9) |
| ^1^ Cerebrovascular accident (n = 2), hyperthyroidism (n = 2), hypothyroidism (n = 2), chemical weapon poisoning (n = 1), scleroderma (n = 1), serious psychiatric disorder (n = 1).  ^2^ Hoarseness (n = 2), fatigue (n = 1).  Abbreviations: SD, standard deviation. | |

| **Supplementary table 2.** Diagnosis and treatment characteristics | |
| --- | --- |
| Variable | Frequency (%) |
| Diagnostic tool  Bronchoscopy  CT-guided biopsy  Surgery  Lymph node biopsy  pleuroscopy | 37 (41.6)  39 (43.8)  6 (6.7)  6 (6.7)  1 (1.1) |
| Pathology  Adenocarcinoma  SCC  SCLC  Carcinoid tumor  Undifferentiated carcinomas | 36 (40.4)  22 (24.7)  17 (19.1)  2 (2.2)  12 (13.5) |
| Stage  Ib  IIb  IIIa  IIIb  IVa  IVb | 1 (1.1)  1 (1.1)  2 (2.2)  13 (14.6)  24 (27.0)  48 (53.9) |
| Treatment  Chemotherapy  Radiotherapy  Surgery  Surgery and chemotherapy  Untreated | 48 (53.9)  30 (33.7)  8 (9.0)  1 (1.1)  2 (2.2) |
| Abbreviations: SCC, squamous cell carcinoma; SCLC, small cell lung cancer. | |
